# COVID-19 symptoms and compliance: The mediating role of fundamental social motives

**DOI:** 10.3389/fpsyg.2023.1093875

**Published:** 2023-03-20

**Authors:** Ruoting Liu, Xueying Zheng, Ziyu Wang, Mingjie Zhou, Jianping Weng, Yan-mei Li, Xuefeng Chen

**Affiliations:** ^1^Key Laboratory of Behavioral Science, Institute of Psychology, Chinese Academy of Sciences (CAS), Beijing, China; ^2^Department of Psychology, University of Chinese Academy of Sciences, Beijing, China; ^3^Department of Endocrinology, The First Affiliated Hospital of University of Science and Technology of China Anhui Provincial Hospital, Hefei, Anhui Province, China; ^4^Department of Life Sciences and Medicine, University of Science and Technology of China, Hefei, Anhui Province, China; ^5^Department of Linguistics and Cognitive Science, College of Arts and Sciences, University of Delaware, Newark, DE, United States; ^6^Key Laboratory of Mental Health, Institute of Psychology, Chinese Academy of Sciences (CAS), Beijing, China; ^7^Institute of Psychology, Chinese Academy of Sciences (CAS), Beijing, China

**Keywords:** COVID-19 symptoms, fundamental social motives, compliance, infectious disease, group norms

## Abstract

**Background:**

Understanding the compliance of infected individuals and the psychological process underlying compliance during pandemics is important for preventing and controlling the spread of pathogens. Our study investigated whether fundamental social motives mediate the relationship between having infectious disease and compliance.

**Methods:**

An online survey was conducted in March 2020, during the severe phase of the COVID-19 outbreak in China to collect data from 15,758 participants. The survey comprised self-report questionnaires with items pertaining to current symptoms (COVID-19 symptoms, other symptoms or no symptoms), the Fundamental Social Motive Inventory, and measures of compliance. Correlation analysis, linear regression analysis, and structural equation model were used for data analysis.

**Results:**

The participants with COVID-19 symptoms had lower levels of compliance than those without symptoms, and their lower compliance was caused by a decrease in disease avoidance (indirect effect = −0.058, 95% CI = [−0.061, −0.056]) and familial motives (indirect effect = −0.113, 95% CI = [−0.116, −0.062]). Whereas exclusion concern (indirect effect = 0.014, 95% CI = [0.011, 0.017]) suppressed the effects of COVID-19 symptoms on compliance, the effect disappeared in the multiple mediation model, while those of disease avoidance and familial motives remained.

**Conclusion:**

Our findings emphasize the critical role of disease avoidance and familial motives in promoting compliance with public health norms during pandemics and suggest that enhancing these motives may serve as an effective intervention strategy to mitigate noncompliance among potentially infected individuals.

## 1. Introduction

The Coronavirus Disease 2019 (COVID-19) pandemic, which emerged in December 2019, rapidly disseminated worldwide (Jin et al., 2020; Park et al., 2020), causing billions of infections and millions of deaths (World Health Organization, 2022). Although the pandemic is gradually abating, the valuable lessons learned and warnings issued by this global crisis endure. In the absence of vaccines, it is crucial to employ effective measures to control the transmission of infectious diseases. A vital strategy for achieving this is by preventing potential carriers of the disease from interacting with others and directing them toward receiving proper diagnosis and treatment (Rothan and Byrareddy, 2020). Despite significant efforts to control the spread of COVID-19, numerous news reports since the onset of the pandemic have indicated that some individuals infected with the virus exhibit noncompliance with recommended health protocols. This includes refusing to quarantine, maintaining social distance, or concealing symptoms to participate in social gatherings, which exacerbates the transmission of the disease. Therefore, the present study aims to investigate whether individuals’ perception of their infectious disease symptoms affects their compliance with group norms during pandemics. Specifically, we hope to explore whether individuals with infectious disease symptoms exhibit lower levels of compliance than those without such symptoms. Fundamental social motives have been considered critical for understanding and predicting people’s behavior in social group living (Jonason and Zeigler-Hill, 2018). Thus, we attempt to examine the effect of infectious symptoms on compliance through the fundamental social motive lens.

### 1.1. COVID-19 symptoms and compliance

Infectious disease has imposed a substantial threat to human survival and reproduction throughout the evolutionary process (Inhorn and Brown, 1990). Salient group norms during pandemics are highly associated with preventing and containing the spread of pathogens. Complying with group norms is beneficial for decreasing infection risk and improving the survival of individuals and groups (Murray et al., 2011; Wu and Chang, 2012). People are more likely to comply with group norms during pandemics (Cashdan and Steele, 2013). However, infected people have been found to have tendencies to deviate from norms. Individuals with COVID-19 symptoms have lower rates of self-isolation and timely testing (Rubin et al., 2020; Smith et al., 2020a,b). Such discrepancy in compliance between infected and uninfected people might be associated with changes in fundamental social motives.

### 1.2. Fundamental social motives in pandemics

Fundamental social motives shaped by human evolutionary history guide behavior to manage recurrent threats, challenges, and opportunities in social life to achieve survival and reproductive goals (Kenrick et al., 2010a). Such systems involve self-protection, disease avoidance, affiliation, status seeking, mate seeking, mate retention and kin care (Neel et al., 2016). The motivational priorities vary with situational cues and individual differences (Griskevicius and Kenrick, 2013; Jonason and Zeigler-Hill, 2018).

The pandemic immensely threatens human survival and genetic continuity (Pyszczynski et al., 2021), and people respond by calibrating their effort distribution to social goals that better enable them to manage threats. In the critical period of pandemics, public health measures (e.g., social distance, stay at home, etc.) greatly limit interpersonal contact and social gatherings, leave little risk of being attacked by others and fewer opportunities for status seeking, mate seeking and establishing/improving affiliations, which reduces the necessity of fulfilling these goals. However, disease avoidance and familial motives [i.e., mate retention, kin care (family) and kin care (children)] may become more important for navigating the challenges in social life during pandemics for people with and without infection symptoms.

Studies have found that the presence of pathogens leads people to perceive themselves as less social and increases their tendency to avoid others (Mortensen et al., 2010). A considerable body of evidence shows that proactive prevention and avoidance behavior greatly reduce infection risk and increase the survival opportunities for individuals and their family members during pandemics (Hatchett et al., 2007; Chan et al., 2020). These findings suggest that the disease avoidance motive directly guides preventive perceptions and behavior during pandemics. Familial motives are generally prioritized in social life (Ko et al., 2020). People prefer to stay with family members or mates when facing threats and fears (Florian et al., 2002; Cox et al., 2008) since genetically related people and mates provide more social support to buffer psychological distress (Li et al., 2021) and exhibit more altruistic behavior toward each other (Acevedo et al., 2019). An article reviewing 45 relevant studies showed that kin contribute to child care and children survival success (Sear and Mace, 2008). This evidence indicates that disease avoidance and familial goals are prioritized in shaping social behavior during pandemics. Salient group norms during pandemics usually aim at preventing and containing infection to reduce the mortality of group members. Compliance with group norms reduces infection risk and improves the survival of individuals and their family members. Disease avoidance and familial motives may facilitate compliance during pandemics.

### 1.3. Infection, compliance and fundamental social motives

Infectious diseases are closely associated with death and exclusion in evolutionary history.

In the early stages of pandemics, death looms larger due to lack of antiviral drugs. Individuals with infection symptoms may be more conscious of the threat of death and have higher death anxiety. As argued by terror management theory, proximal defences are activated to suppress death-related thoughts to reduce death anxiety when conscious of the threat of death (Kosloff et al., 2019). Studies have shown that death anxiety caused an avoidance of threat-related information (Golman et al., 2017; Lee and Kim, 2021), diversion-seeking behavior (Wittkowski, 2015), and avoidance coping strategies during the COVID-19 pandemic (Partouche-Sebban et al., 2022). Denying threat or avoiding risk information about infectious disease decreases the importance and attention to the disease avoidance goal, reducing the disease avoidance motive of individuals with infection symptoms.

Moreover, coping with death anxiety consumes cognitive resources (Hayes et al., 2010), which may lead individuals with infection symptoms to pay less attention to others, even their family members and children. Previous studies have shown that high anxiety is associated with more concern for the self and fewer helping behavior (Mor and Winquist, 2002; Calderwood et al., 2018), making parents pay less attention to their children (Chemtob et al., 2010; Sprang and Silman, 2013). Hence, individuals with infection symptoms may have fewer familial motives.

In conclusion, the perception of having an infectious disease during a pandemic arouses death anxiety, which distracts people from disease prevention and familial relationships. Decreasing disease avoidance and familial motives decrease the necessity and urgency to comply with group norms. Therefore, we hypothesize that disease avoidance and familial motives mediate the relationship between infection symptoms (have vs. not) and compliance during pandemics.

On the other hand, people often label and exclude those with infectious disease (Park et al., 2003), which may initiate the exclusion concern of individuals with infectious symptoms. Social exclusion obstructs access to resources, threatens survival, and has negative impacts on physical and mental health (Macdonald and Leary, 2005). Gaining social acceptance through compliance is a common strategy to alleviate the exclusion concern when perceiving an exclusion threat (Romero-Canyas et al., 2010). Alternatively, concealing a stigma to avoid exclusion is another option but requires cognitive efforts (Meyer, 2003). Managing the threat of death in highly stressful situations costs cognitive resources (Hayes et al., 2010), suggesting that individuals with infection symptoms are more likely to choose a compliance strategy to relieve exclusion concerns. However, individuals with COVID-19 symptoms were found to be less willing to comply with social norms (Rubin et al., 2020; Smith et al., 2020a,b). Thus, we expect the relationship between infection symptoms (have vs. not) and compliance to be suppressed by the exclusion concern motive.

### 1.4. The current study

**Figure 1:**
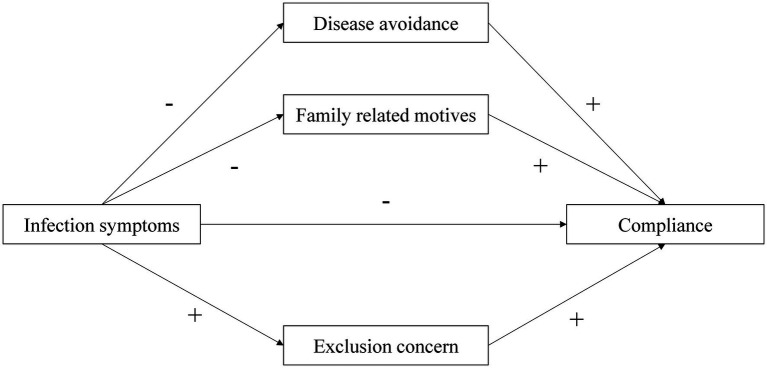
The proposed mediation model.

The primary objective of this study was to examine the effect of infection symptoms on compliance with group norms during a pandemic and the potential effects of fundamental social motives to bias the compliance of individuals exhibiting such symptoms. We explore the mediating effects of disease avoidance and familial motives, as well as the suppression effect of exclusion concern motive on the relationship in both simple and multiple mediation models (Figure 1). The present study contributes to the enhanced comprehension and amelioration of the conduct of infected individuals during pandemics and provides substantial insights for devising public policy and formulating responses to future crises.

## 2. Methods

### 2.1. Participants and procedures

Data were collected from the First Affiliated Hospital of the University of Science and Technology of China online data platform, Risk assessment of 2019-nCoV infection, from March 25 to April 1, 2020. During that period, Wuhan was still under lockdown, and specific antiviral drugs and vaccines against COVID-19 had not yet been developed. The final sample consisted of 15,758 participants (4,999 were male, 31.72%), aged from 17 to 80 (*M_age_* = 41.32 ± 14.216), mostly from Anhui Province (98.97%). All participants participated and completed the questionnaire voluntarily. The study protocol was approved by the institutional review board of the Ethics Committee of the Institute of Psychology, Chinese Academy of Sciences (reference number: H20016).

### 2.2. Measures and materials

#### 2.2.1. Fundamental social motives

The Chinese version of the Fundamental Social Motives Scale was adapted from The Fundamental Social Motives Inventory (Neel et al., 2016) by three social psychologists familiar with the fundamental social motive framework. Each motive was condensed into a single item using the most culturally acceptable expression according to the definition of motives. This scale contains 11 items that assess 11 motives, including disease avoidance, mate retention, kin care (family), kin care (children), exclusion concern, group, friendship retention, independence, self-protection, status, and mate seeking (see Supplementary Table S1 in Additional file 1 for details). Participants reported their current level of intensity toward these motives/goals on a 5-point Likert-type scale ranging from 1 (not at all strong) to 5 (very strong). Cronbach’s Alpha showed a reliability coefficient of 0.745 in this study, while the KMO test value was 0.812 (>0.7), and the Bartlett’s spherical test value was 13,914.080 (*p* < 0.001), indicating good structural validity of the scale.

#### 2.2.2. Compliance

To minimize potential pressure arising from questionnaire completion in the epidemic environment, a comprehensive item was utilized to assess participants’ compliance with group norms. Participants were asked to rate their level of willingness to comply with norms within their *Qunti/Zuzhi*^1^ on a 5-point scale, ranging from 1 (not at all strong) to 5 (very strong).

#### 2.2.3. COVID-19 symptoms

To explore the hypothesis, a question was used to collect information about the participants’ COVID-19 symptoms. The participants were requested to indicate their current symptoms by selecting from the following three options: (1) having symptoms, such as fever, cough, fatigue, etc., that are established COVID-19 symptoms (World Health Organization, 2020); (2) having other symptoms, with an additional option to report symptoms not listed; and (3) not experiencing any symptoms. The participants who selected any of the listed symptoms were coded as *COVID-19 symptoms*, and those who chose other symptoms or no symptoms were coded as *other symptoms* or *no symptoms*, respectively. Previous studies have demonstrated that other symptoms (e.g., dizziness, coryza) are positively correlated with higher levels of anxiety, distress and depression, which may influence behaviors (Stanton et al., 2020; Wang et al., 2020). Therefore, to better test the hypothesis and clarify the impact of infectious disease symptoms on compliance, we included all three symptom groups in the subsequent analysis, resulting in a multi-categorical independent variable. To identify motivational and behavioral biases associated with COVID-19 symptoms, we constructed two dummy variables: *COVID-19 symptoms (vs. no symptoms)* and *other symptoms (vs. no symptoms)*, with healthy individuals serving as the baseline. The severity of other symptoms did not significantly impact fundamental social motives and compliance (*p*s > 0.05), so we consolidated all other symptoms into a single group (see Supplementary Table S2 in Additional file 2 for details).

#### 2.2.4. Control variables

Individual differences in social motives can be influenced by various life history factors, such as sex, age, and job status (Neel et al., 2016; Kavaliers et al., 2019). Pregnancy status has also been identified as an important variable that may affect individuals’ behavior during a pandemic (Safi-Keykaleh et al., 2022). Furthermore, previous studies have shown that factors such as disease history, contact with a source of infection, and the cumulative number of confirmed provincial cases can influence people’s perception of the pandemic (Rodriguez-Rey et al., 2020; Brooks et al., 2021; Aminizadeh et al., 2022). Specifically, medical personnel who are at risk of COVID-19, workplace violence, and work overload may be more prone to anxiety and depression, which can impact their behavior (Heidarijamebozorgi et al., 2021; Jamebozorgi et al., 2022; Sheikhbardsiri et al., 2022). We also considered the implicit answering norm of filling out the blank for reporting other symptoms, which may reflect participants’ compliance. Therefore, sex, age, pregnancy status, job status, disease history, exposure to infection, cumulative number of confirmed provincial cases, medical personnel and details of other symptoms were controlled for in the following analysis.

### 2.3. Statistical analysis

*SPSS* version 23.0 was employed to conduct descriptive statistics, bivariate correlations, and linear regressions to examine the relationship between symptom perception, fundamental social motives, and compliance with group norms, as well as to identify which motives are important for compliance during the pandemic. We used confirmatory factor analysis and structural equation model (SEM) using the *lavaan* package for *R* (Rosseel, 2012; R Core Team, 2022). Confirmatory factor analyze was utilized to specify the latent variable (familial motives). The reliability of the scale was evaluated through Cronbach’s alpha and composite reliability (CR), and discriminant validity in SEM analysis was measured through average variance extracted (AVE). The structural equational model (SEM) was used to explore the theorized relationships among variables and assess the mediation models’ fitness. However, the study noted that *χ^2^* is sensitive to sample size, and since the sample size in this study is very large, obtaining accurate model fits becomes difficult (Wen et al., 2004; Bagozzi and Yi, 2012). Therefore, the present study relied mainly on the following indices to assess model fit: absolute fit indices such as root mean square error of approximation (RMSEA; ≤0.08 recommended), goodness of fit index (GFI; ≥0.90 is acceptable), and standardized root mean square residual (SRMR; ≤0.08 recommended), as well as incremental fit indices such as normed fit index (NFI) and comparative fit index (CFI; NFI, CFI ≥ 0.90 is acceptable; Schermelleh-Engel et al., 2003; Kline, 2016). Finally, this study verified the significance of paths and mediation effects through bias-corrected percentile Bootstrap with 5,000 resamples and a confidence interval of 95%.

## 3. Results

### 3.1. Descriptive statistics and correlations

Table 1 summarizes the characteristics of the sample. The descriptive statistics and correlation matrices of the major variables are presented in Table 2. For the participants with no symptoms or COVID-19 symptoms, compliance was motivated by all fundamental social motives except affiliation (independence). For the participants with other symptoms, compliance was motivated by all fundamental social motives except affiliation (independence) and mate seeking (see Supplementary Table S3 in Additional file 3). The results suggested that people believe that compliance helps them approach most fundamental social goals in life.

**Table 1:** Characteristics of the sample (*N* = 15,758).

Variable	Total sample (*N* = 15,758)		No symptom (*n* = 12,531)		Other symptoms (*n* = 2,540)		COVID-19 symptoms (*n* = 687)	
	*N*	%	*N*	%	*N*	%	*N*	%
Sex								
Male	4,999	31.72%	3,835	30.60%	857	33.74%	307	44.69%
Female	10,759	68.28%	8,696	69.40%	1,683	66.26%	380	55.31%
Age (*M* ± *SD*)	41.318 ± 14.216		40.491 ± 13.965		44.417 ± 14.515		44.948 ± 15.457	
Job status								
At work	13,742	87.21%	10,959	87.46%	2,208	86.93%	575	83.70%
Retiree	1,486	9.43%	1,113	8.88%	279	10.98%	94	13.68%
Student	530	3.36%	459	3.66%	53	2.09%	18	2.62%
Medical staff								
Medical staff	390	2.47%	336	2.68%	44	1.73%	10	1.46%
Nonmedical staff	15,368	97.53%	12,195	97.32%	2,496	98.27%	677	98.54%
Pregnant								
Pregnant	66	0.42%	0	0.00%	66	2.60%	0	0.00%
Not pregnant	15,692	99.58%	12,531	100.00%	2,474	97.40%	687	100.00%
Disease history								
Chronic diseases	3,465	21.99%	2,500	19.95%	695	27.36%	270	39.30%
No chronic diseases	12,293	78.01%	10,031	80.05%	1,845	72.64%	417	60.70%
Exposure to infection								
Has been exposed	23	0.15%	17	0.14%	2	0.08%	4	0.58%
Never been exposed	15,719	99.75%	12,513	99.86%	2,532	99.68%	674	98.11%
Not sure	16	0.10%	1	0.0%	6	0.24%	9	1.31%

**Table 2:** Descriptive statistics and bivariate correlations of major variables.

Variables	*M*	SD	95% CI	1	2	3	4	5
No symptom (*n* = 12,531)								
1 Compliance	3.922	0.936	[3.906, 3.939]					
2 Disease avoidance	4.206	0.876	[4.191, 4.222]	0.393^***^				
3 Mate retention	4.130	0.891	[4.115, 4.146]	0.445^***^	0.413^***^			
4 Kin care (family)	4.234	0.820	[4.220, 4.248]	0.480^***^	0.441^***^	0.563^***^		
5 Kin care (children)	4.144	0.969	[4.127, 4.161]	0.424^***^	0.360^***^	0.486^***^	0.604^***^	
6 Exclusion concern	2.996	1.014	[2.978, 3.013]	0.073^***^	0.101^***^	0.124^***^	0.107^***^	0.107^***^
COVID-19 symptoms (*n* = 687)								
1 Compliance	3.741	1.010	[3.665, 3.817]					
2 Disease avoidance	4.052	0.940	[3.982, 4.123]	0.308^***^				
3 Mate retention	3.999	0.947	[3.928, 4.069]	0.382^***^	0.364^***^			
4 Kin care (family)	4.096	0.870	[4.031, 4.161]	0.413^***^	0.325^***^	0.556^***^		
5 Kin care (children)	4.045	1.006	[3.970, 4.120]	0.419^***^	0.269^***^	0.482^***^	0.616^***^	
6 Exclusion concern	3.176	0.994	[3.102, 3.251]	0.130^**^	0.115^**^	0.175^***^	0.167^***^	0.190^***^
Other symptoms (*n* = 2,540)								
1 Compliance	3.937	0.917	[3.901, 3.973]					
2 Disease avoidance	4.158	0.874	[4.124, 4.192]	0.401^***^				
3 Mate retention	4.087	0.882	[4.053, 4.121]	0.407^***^	0.371^***^			
4 Kin care (family)	4.189	0.826	[4.157, 4.221]	0.465^***^	0.430^***^	0.524^***^		
5 Kin care (children)	4.150	0.921	[4.114, 4.186]	0.444^***^	0.402^***^	0.490^***^	0.621^***^	
6 Exclusion concern	3.063	1.021	[3.024, 3.103]	0.111^***^	0.048^*^	0.113^***^	0.113^***^	0.124^***^

^*^p < 0.05, ^**^p < 0.01, ^***^p < 0.001.

### 3.2. Linear regression analyses

As expected, compliance was significantly lower in the participants with COVID-19 symptoms than in those with no symptoms (*β* = −0.037, *p* < 0.001). There was no significant difference in compliance between the participants with other symptoms and those with no symptoms (*β* = −0.023, *p* = 0.050). Among the control variables, exposure to infection and job status (among students and retirees) corresponded to decreased compliance, whereas participants who reported details regarding other specific symptoms and those who were pregnant exhibited higher compliance (see Supplementary Table S4 in Additional file 4 for details).

Compared with the participants with no symptoms, those with COVID-19 symptoms had lower levels of disease avoidance (*β* = −0.033, *p* < 0.001), mate retention (*β* = −0.032, *p* < 0.001), kin care (family) (*β* = −0.034, *p* < 0.001) and kin care (children) (*β* = −0.025, *p* = 0.001) and a higher level of exclusion concern (*β* = 0.038, *p* < 0.001). The participants reporting other symptoms also had lower levels of disease avoidance (*β* = −0.053, *p* < 0.001), mate retention (*β* = −0.057, *p* < 0.001), kin care (family) (*β* = −0.060, *p* < 0.001) and kin care (children) (*β* = −0.043, *p* < 0.001) and a higher level of exclusion concern (*β* = 0.044, *p* < 0.001) than the participants with no symptoms. Current symptoms had no significant impact on the motives of self-protection, affiliation (group), affiliation (friendship retention), mate seeking, and status seeking (*p*s > 0.05). Additionally, the results showed that the affiliation (independence) motives of the participants with COVID-19 symptoms and those with other symptoms were higher than those of the participants with no symptoms (*p*s < 0.05), suggesting that poor health may lead people to be less social.

The regression of motives on compliance revealed that disease avoidance (*β* = 0.389, *p* < 0.001), familial motives (mate retention: *β* = 0.437, *p* < 0.001; kin care (family): *β* = 0.473, *p* < 0.001; kin care (children): *β* = 0.438, *p* < 0.001) and exclusion concern (*β* = 0.082, *p* < 0.001) improved compliance. Self-protection, affiliation (group), affiliation (friendship retention), mate seeking, and status seeking also had positive effects on compliance (*p*s < 0.001), but affiliation (independence) did not (*p* > 0.05; see Supplementary Table S4 for details).

**Figure 2:**
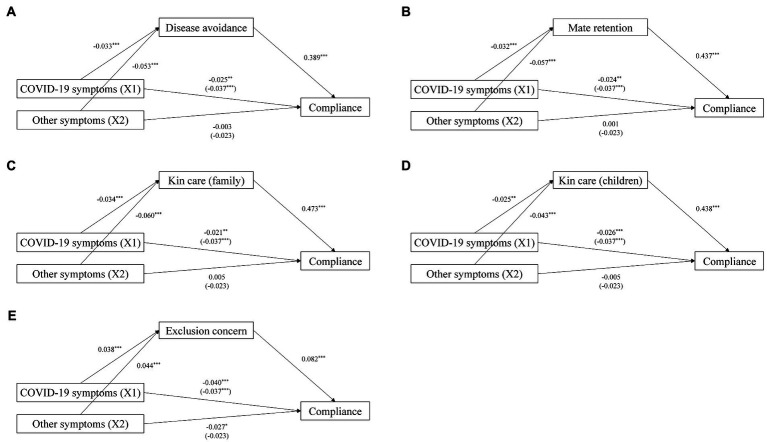
Simple mediation models with standardized path coefficients. *p < 0.05, ^**^p < 0.01, ^***^p < 0.001. Relations among COVID-19 symptoms, **(A)** Disease avoidance and compliance. **(B)** Mate retention and compliance. **(C)** Kin care (family) and compliance. **(D)** Kin care (children) and compliance. **(E)** Exclusion concern and compliance.

These findings indicated that, in line with our predictions, disease avoidance, familial motives and exclusion concern played crucial roles in the link between symptoms and compliance during pandemics. Figure 2 shows the regression results.

### 3.3. Structural equation modeling

A confirmatory factor analysis (CFA) was conducted on the latent variable of familial motives. The analysis demonstrated that the model of the scale had an acceptable fit with the data (CFI = 0.994, GFI = 0.997, NFI = 0.993, SRMR = 0.016, RMSEA = 0.045 [0.039, 0.052]). The standardized factor loading was 0.808 for mate retention, 0.868 for kin care (family) and 0.835 for kin care (children) motives. Additionally, the Cronbach’s *α* was 0.782 (>0.7), CR was 0.784 (>0.6), and AVE was 0.549 (>0.5), suggesting that the measurement model has good reliability and good convergent validity (Bagozzi and Yi, 1988; Hair et al., 2006).

We used SEM to check the fit of predictive mediation model and verify the significance of the indirect effects. As shown in Table 3, Model 1 showed good fit to the data (CFI = 0.902, GFI = 0.985, NFI = 0.901, SRMR = 0.018, RMSEA = 0.051 [0.046, 0.055]). The results suggested that COVID-19 symptoms (vs. no symptoms; relative indirect effect = −0.058, 95% CI = [−0.061, −0.056]), and other symptoms (vs. no symptoms; relative indirect effect = −0.053, 95% CI = [−0.077, −0.028]) indirectly affected compliance *via* disease avoidance motive. Model 2 showed the goodness of model fit as the values of CFI = 0.966, GFI = 0.979, NFI = 0.965, SRMR = 0.012, RMSEA = 0.041 [0.038, 0.043], and suggested that COVID-19 symptoms (vs. no symptoms; relative indirect effect = −0.113, 95% CI = [−0.116, −0.062]) and other symptoms (vs. no symptoms; relative indirect effect = −0.112, 95% CI = [−0.156, −0.072]) indirectly affected compliance *via* familial motives. The fit of Model 3 was not acceptable according to the following parameter: CFI = 0.409, GFI = 0.985, NFI = 0.426, SRMR = 0.018, RMSEA = 0.048 [0.044, 0.052]. However, the relative indirect effects of exclusion concern were significant (COVID-19 symptoms (vs. no symptoms): relative indirect effect = 0.014, 95% CI = [0.011, 0.017]); other symptoms (vs. no symptoms; relative indirect effect = 0.009, 95% CI = [0.004, 0.014]). Given the two relative total effects of Model 3 were negative, exclusion concerns had a suppression effect in this model.

**Table 3:** Model fit indices for SEM of mediation analysis.

SEM model	Path	Relative indirect effect	Boot SE	95% BootCI	CFI	GFI	NFI	RMSEA [90%CI]	SRMR
Model 1	X1-DA-C	−0.058	0.001	[−0.061, −0.056]	0.902	0.985	0.901	0.051 [0.046, 0.055]	0.018
	X1-DA-C	−0.053	0.013	[−0.077, −0.028]					
Model 2	X1-FM-C	−0.113	0.026	[−0.116, −0.062]	0.966	0.979	0.965	0.041 [0.038, 0.043]	0.012
	X2-FM-C	−0.112	0.021	[−0.156, −0.072]					
Model 3	X1-EC-C	0.014	0.001	[0.011, 0.017]	0.409	0.985	0.426	0.048 [0.044, 0.052]	0.018
	X2-EC-C	0.009	0.003	[0.004, 0.014]					
Model 4	X1-DA-C	−0.014	0.004	[−0.023, −0.007]	0.970	0.984	0.969	0.040 [0.038, 0.043]	0.011
	X2-DA-C	−0.013	0.003	[−0.020, −0.007]					
	X1-FM-C	−0.104	0.024	[−0.152, −0.057]					
	X2-FM-C	−0.103	0.020	[−0.144, −0.066]					
	X1-EC-C	−0.002	0.001	[−0.005, 0.000]					
	X2-EC-C	−0.001	0.001	[−0.003, 0.000]					

X1: COVID-19 symptoms (vs. no symptoms); X2: other symptoms (vs. no symptoms); DA: disease avoidance; FM: familial motives; EC: exclusion concern; C: compliance. CFI, GFI, NFI ≥ 0.90 is acceptable; RMSEA ≤ 0.08 recommended; CI = confidence interval; SRMR ≤ 0.08 recommended.

To estimate the specific indirect effect of each mediator while controlling for others, we employed a multiple mediation model. The results show that the model has acceptable fit indices: CFI = 0.970, GFI = 0.984, NFI = 0.969, SRMR = 0.011, RMSEA = 0.040 [0.038, 0.043]. As shown in Table 3, the specific indirect effects are significant for disease avoidance, mate retention, kin care (family), and kin care (children) but not for exclusion concerns. The unique effect of exclusion concern disappeared after controlling for disease avoidance and familial motives. These findings suggest that disease avoidance and familial motives, instead of exclusion concerns, play essential roles in the effects of COVID-19 symptoms on compliance.

In addition, our analysis revealed that age exhibited a positive moderating effect on the pathway from COVID-19 symptoms (vs. no symptoms) to familial motives (*β* = 0.002, *p* = 0.014), suggesting that age alleviated the negative effect of COVID-19 symptoms on familial motives. As noted by the life history theory, age can serve as a proxy for life stage, and with advancing age, individuals tend to shift their focus from somatic effort to reproductive effort (Neel et al., 2016). To ensure successful reproduction and offspring rearing, people tend to prioritize investing in kinship and mate relationships, leading to the strengthening and stability of familial motives across the adult lifespan (Finkel and Eastwick, 2015; Ko et al., 2020).

## 4. Discussion

In the early stages of pandemics, specific antiviral drugs and vaccines are not available to the general public, and group norms become crucial in preventing the spread of diseases. The current study investigated the compliance of individuals with infectious symptoms and whether fundamental social motives mediate the relationship between infectious symptoms and compliance through a large sample survey conducted during the severe period of the COVID-19 outbreak in China.

### 4.1. Symptoms, fundamental social motives and compliance

#### 4.1.1. Individuals with COVID-19 symptoms

In line with our expectation, the individuals with COVID-19 symptoms complied less with group norms than those with no symptoms, which can be attributed to the decrease in disease avoidance and familial motives. A reasonable explanation is that death anxiety interferes with the fundamental social motives that are important for shaping compliance behavior during pandemics. Individuals who perceive themselves to have COVID-19 symptoms experience higher death anxiety (Sadri Damirchi et al., 2020; Zeng et al., 2021), which results in less attention given to risk information related to infectious disease and less concern for others, thereby decreasing disease avoidance and familial motives. Our results are broadly consistent with the findings of research on COVID-19, such as death anxiety leading to denial of the virus’s contagiousness or lethality and the avoidance of important information (Menzies and Menzies, 2020; Song et al., 2021; Partouche-Sebban et al., 2022) and COVID-19 symptoms undermining the ability to care for family members and children (Spinelli et al., 2020; Ding et al., 2021).

As expected, exclusion concerns suppressed the effects of infectious symptoms on compliance, suggesting that increases in exclusion concerns decreased the compliance of infected individuals to some extent. The behavioral immune system leads people to prevent infection through aversion, exclusion or avoidance of infected individuals (Murray and Schaller, 2016). When feeling a threat of exclusion, individuals with infectious symptoms may attempt to obtain social acceptance through compliance. Nevertheless, the suppression effect disappeared with the mediation effects of disease avoidance and familial motives remained in the multiple mediation model. The indirect effect may have attenuated because disease avoidance and familial motives are more important for managing threats and the chance to live through a pandemic society than exclusion concerns, even for individuals with infectious symptoms. This is supported by our results that the disease avoidance and familial motives of individuals with or without COVID-19 symptoms were significantly higher than their exclusion concerns (*p*s < 0.001, see Table 2 for details).

#### 4.1.2. Individuals with other symptoms

Additionally, we found that the compliance of individuals with other symptoms decreased with the reduction in disease avoidance and familial motives, although the direct effects were not significant. Having other symptoms indicates poor health, which increases the perceived risk of COVID-19 infection and death anxiety (Stanton et al., 2020). As a result, attention to disease avoidance and familial care should be decreased, but the insignificant direct effects suggest that the impacts of other symptoms on compliance are subtle, and other factors may play a part in the relationship (Shrout and Bolger, 2002). Perceived vulnerability to disease (PVD) predicts conformity attitudes (Wu and Chang, 2012). During the COVID-19 pandemic, PVD was found to increase preventive behavior and beliefs in public health measures, whereas anxiety and depression increase maladaptive behavior (De Coninck et al., 2020; Stangier et al., 2021). These findings indicate that PVD and anxiety may influence the relationship between other diseases and compliance in different directions. Future research should explore how PVD intertwines with anxiety to affect the compliance of individuals with other diseases during pandemics.

### 4.2. Theoretical and practical implications

A large sample survey conducted during the critical period of the COVID-19 pandemic to explore the social motive mechanism underlying the compliance of people with infectious disease has strong ecological validity and important implications for both understanding the psychological processes of infected people and containing the spread of infectious disease.

In line with previous studies (Rubin et al., 2020; Smith et al., 2020a,b), the current study showed that people with infectious disease complied less with norms and went further by showing that having infectious disease or a poor health status affects compliance *via* multiple fundamental social motives. These findings are consistent with studies showing that individual differences influence the priority of social motives and that motives guide behavior (Maner et al., 2005; Kenrick et al., 2010b). Different from past works focusing mainly on life history variables (e.g., sex, age, relationship status; Finkel and Eastwick, 2015; Jonason and Zeigler-Hill, 2018), our study demonstrated that the perception of having an infectious disease and health status are also important factors predicting changes in social motives, which ultimately affect compliance during pandemics.

The second implication lies in the finding that reduced disease avoidance and familial motives biased the compliance of infected people and those with poor health during pandemics, which highlights the motivational bias of people at high risk to comply with norms that help prevent and contain the infectious disease. These findings indicate that interventions aimed at increasing these motives might serve as an effective strategy to improve compliance during a public health crisis such as the COVID-19 pandemic. For instance, media advocacy and effective hospital policies can guide the motive levels of infected individuals toward improving their behavior (Sheikhbardsiri et al., 2020; Chen et al., 2022).

### 4.3. Limitations and future directions

The first limitation is that to minimize the length of the questionnaire for application during the pandemic, the study included only one measure of compliance. Previous studies showed that compliance tendency varies with the type of norms. For example, compliance with cleaning behavior was higher than compliance with containing behavior during the COVID-19 pandemic (Toussaint et al., 2020; Bailey et al., 2021). Thus, in future studies, we plan to develop graphical scenarios of compliance to investigate whether the effects of the perception of having an infectious disease on compliance vary across norms. Similarly, to alleviate the burden on study participates, single-item and comprehensive measures of each motive were employed in this study. While the overall reliability of the Fundamental Social Motives Scale is deemed satisfactory, using a multiple-item scale may be more advantageous for measuring specific motives. Thus, in future studies, we aim to provide more detailed measures of motives.

Second, previous studies have shown that the perception of having COVID-19 symptoms increases death anxiety (Sadri Damirchi et al., 2020), but the current study did not measure death anxiety directly. Future research should verify the role of death anxiety in the relationship between the perception of having an infectious disease and fundamental social motives.

Third, the data in this study are cross-sectional in nature, which prevents causal claims from being supported. Future research should manipulate the perception of having an infectious disease as well as the pandemic threat to test the causal links between infectious disease and fundamental social motives and the links between infectious disease and compliance by experiments. However, as an exploratory investigation during a real pandemic, the study provides a new perspective and important preliminary findings for understanding the mechanism underlying the compliance of people with infectious disease.

## 5. Conclusion

The present study shows that infectious disease symptoms predict compliance with group norms during pandemics and that multiple fundamental social motives mediate the relationship. Having COVID-19 symptoms leads to lower compliance with group norms mainly due to a reduction in disease avoidance and familial motives. Whereas exclusion concern has a suppression effect on the relationship between having COVID-19 symptoms and compliance, the effect disappeared when disease avoidance and familial motives were controlled for in the multiple mediation model. These findings demonstrate that disease avoidance and familial motives play essential roles in the compliance of individuals with infectious disease during pandemics. Our findings highlight the importance of taking fundamental social motives into consideration when developing interventions to control the spread of pathogens in public health crises during pandemics.

## Data availability statement

The original contributions presented in the study are included in the article/Supplementary material, further inquiries can be directed to the corresponding authors.

## Ethics statement

The studies involving human participants were reviewed and approved by the Ethics Committee of Institute of Psychology, Chinese Academy of Sciences. The patients/participants provided their written informed consent to participate in this study.

## Author contributions

Y-mL and XC provided guidance in the study design and are the corresponding authors. RL and XZ carried out data analysis and wrote the paper. ZW helped with the data analysis, interpretation and write-up. MZ and JW contributed to the data collection. All authors contributed to the article and approved the submitted version.

## Funding

The current research was supported by the National Social Science Foundation of China (grant no. 19ZDA358) and the Scientific Foundation of Institute of Psychology, Chinese Academy of Sciences (no. E2CX3315CX).

## Conflict of interest

The authors declare that the research was conducted in the absence of any commercial or financial relationships that could be construed as a potential conflict of interest.

## Publisher’s note

All claims expressed in this article are solely those of the authors and do not necessarily represent those of their affiliated organizations, or those of the publisher, the editors and the reviewers. Any product that may be evaluated in this article, or claim that may be made by its manufacturer, is not guaranteed or endorsed by the publisher.

## Footnotes

^1^In Chinese, *Qunti* is translated as *group* and *Zuzhi* is translated as *organization*. Although *organization* is a type of *group*, our preliminary study found that lay persons in China believe that *Zuzhi* is different from *Qunti* (1 = not at all, 10 = very different; *M* = 7.43), *Zuzhi* are generally considered a task group with high entitativity, like governmental agencies, political parties (particularly the Communist Party of China), and affiliations associated with work and study (e.g., company, university and school classes), while *Qunti* are considered loose associations and social categories (e.g., women, college students). Thus, we parallel *Qunti* and *Zuzhi* in the question to express the concept of group.

## References

[ref1] Acevedo B. P. Poulin M. J. Brown L. L. (2019). Beyond romance: neural and genetic correlates of altruism in pair-bonds. Behav. Neurosci. 133, 18–31. doi: 10.1037/bne0000293, PMID: 30688485

[ref2] Aminizadeh M. Saberinia A. Salahi S. Sarhadi M. Jangipour Afshar P. Sheikhbardsiri H. (2022). Quality of working life and organizational commitment of Iranian pre-hospital paramedic employees during the 2019 novel coronavirus outbreak. Int. J. Healthcare Manage. 15, 36–44. doi: 10.1080/20479700.2020.1836734

[ref3] Bagozzi R. P. Yi Y. (1988). On the evaluation of structural equation models. J. Acad. Mark. Sci. 16, 74–94. doi: 10.1007/BF02723327

[ref4] Bagozzi R. P. Yi Y. (2012). Specification, evaluation, and interpretation of structural equation models. J. Acad. Mark. Sci. 40, 8–34. doi: 10.1007/s11747-011-0278-x

[ref5] Bailey B. Whelen M. L. Strunk D. R. (2021). Adhering to COVID-19 health guidelines: examining demographic and psychological predictors of adherence. Appl. Psychol.-Health Well Being 13, 968–985. doi: 10.1111/aphw.12284, PMID: 34032383 PMC8239601

[ref6] Brooks S. K. Greenberg N. Wessely S. Rubin G. J. (2021). Factors affecting healthcare workers' compliance with social and behavioral infection control measures during emerging infectious disease outbreaks: rapid evidence review. BMJ Open 11:e049857. doi: 10.1136/bmjopen-2021-049857, PMID: 34400459 PMC8370838

[ref7] Calderwood C. Bennett A. A. Gabriel A. S. Trougakos J. P. Dahling J. J. (2018). Too anxious to help? Off-job affective rumination as a linking mechanism between work anxiety and helping. J. Occup. Organ. Psychol. 91, 681–687. doi: 10.1111/joop.12220

[ref8] Cashdan E. Steele M. (2013). Pathogen prevalence, group bias, and collectivism in the standard cross-cultural sample. Hum. Nat. 24, 59–75. doi: 10.1007/s12110-012-9159-3, PMID: 23389437

[ref9] Chan J. F.-W. Yuan S. Kok K.-H. To K. K. W. Chu H. Yang J.. (2020). A familial cluster of pneumonia associated with the 2019 novel coronavirus indicating person-to-person transmission: a study of a family cluster. Lancet 395, 514–523. doi: 10.1016/s0140-6736(20)30154-9, PMID: 31986261 PMC7159286

[ref10] Chemtob C. M. Nomura Y. Rajendran K. Yehuda R. Schwartz D. Abramovitz R. (2010). Impact of maternal posttraumatic stress disorder and depression following exposure to the September 11 attacks on preschool children's behavior. Child Dev. 81, 1129–1141. doi: 10.1111/j.1467-8624.2010.01458.x, PMID: 20636686 PMC3124807

[ref01] Chen X. Liu T. Li P. Wei W. Chao M. (2022). The relationship between media involvement and death anxiety of self-quarantined people in the COVID-19 outbreak in China: The mediating roles of empathy and sympathy. Omega 85, 974–989. doi: 10.1177/0030222820960283, PMID: 32955991 PMC9361034

[ref11] Cox C. R. Arndt J. Pyszczynski T. Greenberg J. Abdollahi A. Solomon S. (2008). Terror management and adults' attachment to their parents: the safe haven remains. J. Pers. Soc. Psychol. 94, 696–717. doi: 10.1037/0022-3514.94.4.696, PMID: 18361679

[ref13] De Coninck D. d’Haenens L. Matthijs K. (2020). Perceived vulnerability to disease and attitudes towards public health measures: COVID-19 in Flanders, Belgium. Pers. Individ. Diff. 166:110220. doi: 10.1016/j.paid.2020.110220, PMID: 32834279 PMC7327450

[ref14] Ding Y. Ji T. Guo Y. (2021). Helping while social distancing: pathogen avoidance motives influence People’s helping intentions during the COVID-19 pandemic. Int. J. Environ. Res. Public Health 18:12113. doi: 10.3390/ijerph182212113, PMID: 34831871 PMC8622284

[ref15] Finkel E. J. Eastwick P. W. (2015). Attachment and pairbonding. Curr. Opin. Behav. Sci. 3, 7–11. doi: 10.1016/j.cobeha.2014.12.006

[ref16] Florian V. Mikulincer M. Hirschberger G. (2002). The anxiety-buffering function of close relationships: evidence that relationship commitment acts as a terror management mechanism. J. Pers. Soc. Psychol. 82, 527–542. doi: 10.1037//0022-3514.82.4.527, PMID: 11999922

[ref17] Golman R. Hagmann D. Loewenstein G. (2017). Information avoidance. J. Econ. Lit. 55, 96–135. doi: 10.1257/jel.20151245

[ref18] Griskevicius V. Kenrick D. T. (2013). Fundamental motives: how evolutionary needs influence consumer behavior. J. Consum. Psychol. 23, 372–386. doi: 10.1016/j.jcps.2013.03.003

[ref19] Hair J. F. Black W. C. Babin B. J. Anderson R. E. Tatham R. L. (2006). Multivariate data analysis. Hoboken, NJ: Prentice Hall.

[ref20] Hatchett R. J. Mecher C. E. Lipsitch M. (2007). Public health interventions and epidemic intensity during the 1918 influenza pandemic. Proc. Natl. Acad. Sci. U. S. A. 104, 7582–7587. doi: 10.1073/pnas.0610941104, PMID: 17416679 PMC1849867

[ref22] Hayes J. Schimel J. Arndt J. Faucher E. H. (2010). A theoretical and empirical review of the death-thought accessibility concept in terror management research. Psychol. Bull. 136, 699–739. doi: 10.1037/a0020524, PMID: 20804234

[ref23] Heidarijamebozorgi M. Jafari H. Sadeghi R. Sheikhbardsiri H. Kargar M. Gharaghani M. A. (2021). The prevalence of depression, anxiety, and stress among nurses during the coronavirus disease 2019: a comparison between nurses in the frontline and the second line of care delivery. Nursing Midwifery Stud. 10, 188–193. doi: 10.4103/nms.nms_103_20

[ref25] Inhorn M. C. Brown P. J. (1990). The anthropology of infectious disease. Annu. Rev. Anthropol. 19, 89–117. doi: 10.1146/annurev.an.19.100190.000513

[ref26] Jamebozorgi M. H. Karamoozian A. Bardsiri T. I. Sheikhbardsiri H. (2022). Nurses burnout, resilience, and its association with socio-demographic factors during COVID-19 pandemic. Front. Psych. 12:803506. doi: 10.3389/fpsyt.2021.803506, PMID: 35095618 PMC8795765

[ref27] Jin Y. H. Cai L. Cheng Z. S. Cheng H. Deng T. Fan Y. P.. (2020). A rapid advice guideline for the diagnosis and treatment of 2019 novel coronavirus (2019-nCoV) infected pneumonia (standard version). Mil. Med. Res. 7:4. doi: 10.1186/s40779-020-0233-6 32029004 PMC7003341

[ref28] Jonason P. K. Zeigler-Hill V. (2018). The fundamental social motives that characterize dark personality traits. Personal. Individ. Differ. 132, 98–107. doi: 10.1016/j.paid.2018.05.031

[ref29] Kavaliers M. Ossenkopp K. P. Choleris E. (2019). Social neuroscience of disgust. Genes Brain Behav. 18:e12508. doi: 10.1111/gbb.12508 30062689

[ref30] Kenrick D. T. Griskevicius V. Neuberg S. L. Schaller M. (2010a). Renovating the pyramid of needs: contemporary extensions built upon ancient foundations. Perspect. Psychol. Sci. 5, 292–314. doi: 10.1177/1745691610369469, PMID: 21874133 PMC3161123

[ref31] Kenrick D. T. Neuberg S. L. Griskevicius V. Becker D. V. Schaller M. (2010b). Goal-driven cognition and functional behavior: the fundamental-motives framework. Curr. Dir. Psychol. Sci. 19, 63–67. doi: 10.1177/0963721409359281, PMID: 21874097 PMC3161125

[ref32] Kline R. B. (2016). Principles and practice of structural equation modeling. 4th Edn. New York, NY: Guilford Press.

[ref33] Ko A. Pick C. M. Kwon J. Y. Barlev M. Krems J. A. Varnum M. E. W.. (2020). Family matters: rethinking the psychology of human social motivation. Perspect. Psychol. Sci. 15, 173–201. doi: 10.1177/1745691619872986, PMID: 31791196

[ref34] Kosloff S. Anderson G. Nottbohm A. Hoshiko B. (2019). “Proximal and distal terror management defenses: a systematic review and analysis” in Handbook of terror management theory. eds. Routledge C. Vess M. (London: Elsevier Academic Press), 31–63.

[ref35] Lee J. Kim Y. (2021). How terrorism cues affect attitude polarization over undocumented immigrants via negative emotions and information avoidance: a terror management theory perspective. Soc. Sci. J., 1–16. doi: 10.1080/03623319.2021.1884777

[ref36] Li F. Luo S. Mu W. Li Y. Ye L. Zheng X.. (2021). Effects of sources of social support and resilience on the mental health of different age groups during the COVID-19 pandemic. BMC Psychiatry 21:16. doi: 10.1186/s12888-020-03012-1, PMID: 33413238 PMC7789076

[ref37] Macdonald G. Leary M. R. (2005). Why does social exclusion hurt? The relationship between social and physical pain. Psychol. Bull. 131, 202–223. doi: 10.1037/0033-2909.131.2.202, PMID: 15740417

[ref38] Maner J. K. Kenrick D. T. Becker D. V. Robertson T. E. Hofer B. Neuberg S. L.. (2005). Functional projection: how fundamental social motives can bias interpersonal perception. J. Pers. Soc. Psychol. 88, 63–78. doi: 10.1037/0022-3514.88.1.63, PMID: 15631575

[ref39] Menzies R. E. Menzies R. G. (2020). Death anxiety in the time of COVID-19: theoretical explanations and clinical implications. tCBT 13:e19. doi: 10.1017/S1754470X20000215, PMID: 34191938 PMC7308596

[ref40] Meyer I. H. (2003). Prejudice, social stress, and mental health in lesbian, gay, and bisexual populations: conceptual issues and research evidence. Psychol. Bull. 129, 674–697. doi: 10.1037/0033-2909.129.5.674, PMID: 12956539 PMC2072932

[ref41] Mor N. Winquist J. (2002). Self-focused attention and negative affect: a meta-analysis. Psychol. Bull. 128, 638–662. doi: 10.1037/0033-2909.128.4.638, PMID: 12081086

[ref42] Mortensen C. R. Becker D. V. Ackerman J. M. Neuberg S. L. Kenrick D. T. (2010). Infection breeds reticence: the effects of disease salience on self-perceptions of personality and behavioral avoidance tendencies. Psychol. Sci. 21, 440–447. doi: 10.1177/0956797610361706, PMID: 20424082

[ref43] Murray D. R. Schaller M. (2016). “The behavioral immune system: implications for social cognition, social interaction, and social influence” in Advances in experimental social psychology. eds. Olson J. M. Zanna M. P., vol. 53 (London: Elsevier Academic Press Inc.), 75–129.

[ref44] Murray D. R. Trudeau R. Schaller M. (2011). On the origins of cultural differences in conformity: four tests of the pathogen prevalence hypothesis. Personal. Soc. Psychol. Bull. 37, 318–329. doi: 10.1177/0146167210394451, PMID: 21307175

[ref45] Neel R. Kenrick D. T. White A. E. Neuberg S. L. (2016). Individual differences in fundamental social motives. J. Pers. Soc. Psychol. 110, 887–907. doi: 10.1037/pspp0000068 26371400

[ref46] Park J. H. Faulkner J. Schaller M. (2003). Evolved disease-avoidance processes and contemporary anti-social behavior: prejudicial attitudes and avoidance of people with physical disabilities. J. Nonverbal Behav. 27, 65–87. doi: 10.1023/A:1023910408854

[ref47] Park C. L. Russell B. S. Fendrich M. Finkelstein-Fox L. Hutchison M. Becker J. (2020). Americans' COVID-19 stress, coping, and adherence to CDC guidelines. J. Gen. Intern. Med. 35, 2296–2303. doi: 10.1007/s11606-020-05898-9, PMID: 32472486 PMC7259430

[ref48] Partouche-Sebban J. Vessal S. R. Sorio R. Castellano S. Khelladi I. Orhan M. A. (2022). How death anxiety influences coping strategies during the COVID-19 pandemic: investigating the role of spirituality, national identity, lockdown and trust. J. Mark. Manag. 37, 1815–1839. doi: 10.1080/0267257x.2021.2012232

[ref49] Pyszczynski T. Lockett M. Greenberg J. Solomon S. (2021). Terror management theory and the COVID-19 pandemic. J. Humanist. Psychol. 61, 173–189. doi: 10.1177/0022167820959488 PMC7498956 38603072

[ref50] R Core Team (2022). R: A language and environment for statistical computing. R Foundation for Statistical Computing, Vienna, Austria. Available at: https://www.R-project.org/

[ref51] Rodriguez-Rey R. Garrido-Hernansaiz H. Bueno-Guerra N. (2020). Working in the times of COVID-19. Psychological impact of the pandemic in frontline Workers in Spain. Int. J. Environ. Res. Public Health 17:8149. doi: 10.3390/ijerph17218149, PMID: 33158180 PMC7663407

[ref52] Romero-Canyas R. Downey G. Reddy K. S. Rodriguez S. Cavanaugh T. J. Pelayo R. (2010). Paying to belong: when does rejection trigger ingratiation? J. Pers. Soc. Psychol. 99, 802–823. doi: 10.1037/a0020013, PMID: 20649367 PMC2992828

[ref53] Rosseel Y. (2012). Lavaan: an R package for structural equation modeling. J. Stat. Softw. 48, 1–36. doi: 10.18637/jss.v048.i02

[ref54] Rothan H. A. Byrareddy S. N. (2020). The epidemiology and pathogenesis of coronavirus disease (COVID-19) outbreak. J. Autoimmun. 109:102433. doi: 10.1016/j.jaut.2020.102433, PMID: 32113704 PMC7127067

[ref55] Rubin G. J. Smith L. E. Melendez-Torres G. J. Yardley L. (2020). Improving adherence to 'test, trace and isolate'. J. R. Soc. Med. 113, 335–338. doi: 10.1177/0141076820956824, PMID: 32910870 PMC7488807

[ref56] Sadri Damirchi E. Mojarrad A. Pireinaladin S. Grjibovski A. M. (2020). The role of self-talk in predicting death anxiety, obsessive-compulsive disorder, and coping strategies in the face of coronavirus disease (COVID-19). Iran. J. Psychiatry 15, 182–188. doi: 10.18502/ijps.v15i3.3810, PMID: 33193766 PMC7603592

[ref57] Safi-Keykaleh M. Aliakbari F. Safarpour H. Safari M. Tahernejad A. Sheikhbardsiri H.. (2022). Prevalence of postpartum depression in women amid the COVID-19 pandemic: a systematic review and meta-analysis. Int. J. Gynecol. Obstet. 157, 240–247. doi: 10.1002/ijgo.14129, PMID: 35122433 PMC9087783

[ref58] Schermelleh-Engel K. Moosbrugger H. Müller H. (2003). Evaluating the fit of structural equation models: tests of significance and descriptive goodness-of-fit measures. Methods Psychol. Res. 8, 23–74.

[ref59] Sear R. Mace R. (2008). Who keeps children alive? A review of the effects of kin on child survival. Evol. Hum. Behav. 29, 1–18. doi: 10.1016/j.evolhumbehav.2007.10.001

[ref60] Sheikhbardsiri H. Afshar P. J. Baniasadi H. Farokhzadian J. (2022). Workplace violence against prehospital paramedic personnel (City and road) and factors related to this type of violence in Iran. J. Interpers. Violence 37:NP11683–NP11698. doi: 10.1177/0886260520967127, PMID: 33107378

[ref61] Sheikhbardsiri H. Esamaeili Abdar Z. Sheikhasadi H. Ayoubi Mahani S. Sarani A. (2020). Observance of patients’ rights in emergency department of educational hospitals in south-East Iran. Int. J. Hum. Rights Healthcare 13, 435–444. doi: 10.1108/IJHRH-09-2019-0072

[ref62] Shrout P. E. Bolger N. (2002). Mediation in experimental and nonexperimental studies: new procedures and recommendations. Psychol. Methods 7, 422–445. doi: 10.1037//1082-989x.7.4.422, PMID: 12530702

[ref63] Smith L. E. Amlot R. Lambert H. Oliver I. Robin C. Yardley L.. (2020a). Factors associated with adherence to self-isolation and lockdown measures in the UK: a cross-sectional survey. Public Health 187, 41–52. doi: 10.1016/j.puhe.2020.07.024, PMID: 32898760 PMC7474581

[ref64] Smith L. E. Mottershaw A. L. Egan M. Waller J. Marteau T. M. Rubin G. J. (2020b). The impact of believing you have had COVID-19 on self-reported behavior: cross-sectional survey. PLoS One 15:e0240399. doi: 10.1371/journal.pone.0240399, PMID: 33147219 PMC7641362

[ref65] Song S. Yao X. Wen N. (2021). What motivates Chinese consumers to avoid information about the COVID-19 pandemic?: the perspective of the stimulus-organism-response model. Inf. Process. Manag. 58:102407. doi: 10.1016/j.ipm.2020.102407, PMID: 33041437 PMC7536537

[ref66] Spinelli M. Lionetti F. Pastore M. Fasolo M. (2020). Parents' stress and Children's psychological problems in families facing the COVID-19 outbreak in Italy. Front. Psychol. 11:1713. doi: 10.3389/fpsyg.2020.01713, PMID: 32719646 PMC7350926

[ref67] Sprang G. Silman M. (2013). Posttraumatic stress disorder in parents and youth after health-related disasters. Disaster Med. Public Health Prep. 7, 105–110. doi: 10.1017/dmp.2013.22, PMID: 24618142

[ref68] Stangier U. Kananian S. Schüller J. (2021). Perceived vulnerability to disease, knowledge about covid-19, and changes in preventive behavior during lockdown in a german convenience sample. Curr. Psychol. 41, 7362–7370. doi: 10.1007/s12144-021-01456-6, PMID: 33654348 PMC7906828

[ref69] Stanton R. To Q. G. Khalesi S. Williams S. L. Alley S. J. Thwaite T. L.. (2020). Depression, anxiety and stress during COVID-19: associations with changes in physical activity, sleep, tobacco and alcohol use in Australian adults. Int. J. Environ. Res. Public Health 17:4065. doi: 10.3390/ijerph17114065, PMID: 32517294 PMC7312903

[ref70] Toussaint L. L. Cheadle A. D. Fox J. Williams D. R. (2020). Clean and contain: initial development of a measure of infection prevention behaviors during the COVID-19 pandemic. Ann. Behav. Med. 54, 619–625. doi: 10.1093/abm/kaaa064, PMID: 32856691 PMC7499486

[ref02] Wang C. Pan R. Wan X. Tan Y. Xu L. Ho C. S.. (2020). Immediate Psychological Responses and Associated Factors during the Initial Stage of the 2019 Coronavirus Disease (COVID-19) Epidemic among the General Population in China. Int J Environ Res Public Health. 17:1729. doi: 10.3390/ijerph17051729, PMID: 32155789 PMC7084952

[ref71] Wen Z. L. Hau K.-T. Warsh H. W. (2004). Structural equation model testing: cutoff criteria for goodness of fit indices and chi-square test. Acta Psychol. Sin. 02, 186–194.

[ref72] Wittkowski J. (2015). Coping and attitudes toward dying and death in German adults. OMEGA – J. Death Dying 72, 316–339. doi: 10.1177/0030222815575283

[ref73] World Health Organization. (2020.). Coronavirus disease (COVID-19) symptoms. Available at: https://www.who.int/health-topics/coronavirus#tab=tab_3 (Accessed March 3, 2020).

[ref74] World Health Organization. (2022). WHO coronavirus (COVID-19) dashboard. Available at: https://covid19.who.int/ (Accessed August 1, 2022).

[ref75] Wu B.-P. Chang L. (2012). The social impact of pathogen threat: how disease salience influences conformity. Personal. Individ. Differ. 53, 50–54. doi: 10.1016/j.paid.2012.02.023

[ref76] Zeng Q. Cao H. Ma Q. Chen J. Shi H. Li J. (2021). Appetite loss, death anxiety and medical coping modes in COVID-19 patients: a cross-sectional study. Nurs. Open 8, 3242–3250. doi: 10.1002/nop2.1037, PMID: 34463433 PMC8510753

