# Supplementary material for: COVID-19 symptoms and compliance: The mediating role of fundamental social motives
Source: Front Psychol. 2023 Mar 20;14:1093875. doi: 10.3389/fpsyg.2023.1093875 (PMC10067610; doi:10.3389/fpsyg.2023.1093875)
Supplement: Supplementary file 1 [file Data_Sheet_1.ZIP › Additional file 1.docx]

**Additional file 1**

**Table S1** The Chinese short version of the Fundamental Social Motives Scale

| **中文** | **English** |
| --- | --- |
| 1. 保护自己不受其他人类的伤害 | Keep myself safe from others |
| 1. 避开疾病传染 | Avoid the spread of disease |
| 1. 获得并维护友谊 | Obtain and maintain friendships |
| 1. 归属于某个群体 | Be part of a group |
| 1. 获得成功、权力、财富或地位 | Attain success, power, wealth, or status |
| 1. 找伴侣或对象 | Find a romantic or sexual partner |
| 1. 忠诚于自己的伴侣 | Be faithful to my partner |
| 1. 担心被别人排斥 | Worry about being rejected |
| 1. 关爱照顾家人 | Care for family members |
| 1. 养育照顾子女 | Take care of my children |
| 1. 更愿意一个人待着 | Prefer to spend time alone |
