# Supplementary material for: COVID-19 symptoms and compliance: The mediating role of fundamental social motives
Source: Front Psychol. 2023 Mar 20;14:1093875. doi: 10.3389/fpsyg.2023.1093875 (PMC10067610; doi:10.3389/fpsyg.2023.1093875)
Supplement: Supplementary file 1 [file Data_Sheet_1.ZIP › Additional file 2.docx]

**Additional file** **2**

In the *other symptoms* group, 46 participants reported serious illness, such as cancer or tumour, which have been shown in previous studies to cause intense stress and to possibly impact fundamental social motives (Isaksson et al., 2018; Kenrick et al., 2010). Linear regression results indicated that after controlling for other variables, the compliance and fundamental social motive of those with severe other symptoms were not significantly different from those with nonsevere other symptoms (such as dyspepsia, insomnia, etc.; *n* = 2494) (*p*s > 0.05, see Table S2), so we combined them into one group. This may be because during an epidemic, infectious diseases pose a more immediate and intense threat to survival than other diseases (severe or not) (Pyszczynski et al., 2021).

**Table S2** Regression results of the effect of the severity of other symptoms on the main variables

| Dependent variable | Independent variable | *β* | *SE* | *t* | *p* | 95% CI | *R^2^* | *F* |
| --- | --- | --- | --- | --- | --- | --- | --- | --- |
| Compliance | Severity | -0.014 | 0.138 | -0.677 | 0.498 | [-0.449, 0.262] | 0.005 | 2.191^*^ |
| Disease avoidance | Severity | 0.032 | 0.131 | 1.619 | 0.105 | [-0.126, 0.550] | 0.008 | 2.778^**^ |
| Mate retention | Severity | 0.018 | 0.132 | 0.876 | 0.381 | [-0.225, 0.457] | 0.010 | 3.256^***^ |
| Kin care (family) | Severity | 0.028 | 0.124 | 1.408 | 0.159 | [-0.145, 0.493] | 0.010 | 3.304^***^ |
| Kin care (child) | Severity | 0.030 | 0.137 | 1.507 | 0.132 | [-0.146, 0.558] | 0.030 | 8.063^***^ |
| Exclusion concern | Severity | -0.024 | 0.153 | -1.213 | 0.225 | [-0.582, 0.209] | 0.004 | 1.893^*^ |

Note: Severity (nonsevere = 0, severe = 1). *β* = standardized beta. CI = confidence intervals. *^*^p* < 0.05. *^**^p* < 0.01. *^***^p* < 0.001.

# References

1. Isaksson, J., Lilliehorn, S., & Salander, P. (2018). Cancer patients' motives for psychosocial consultation-Oncology social workers' perceptions of 226 patient cases. *Psychooncology*, 27(4), 1180-1184. <https://doi.org/10.1002/pon.4633>
2. Kenrick, D. T., Neuberg, S. L., Griskevicius, V., Becker, D. V., & Schaller, M. (2010). Goal-Driven Cognition and Functional Behavior: The Fundamental-Motives Framework. *Current Directions in Psychological Science*, 19(1), 63-67. <https://doi.org/10.1177/0963721409359281>
3. Pyszczynski, T., Lockett, M., Greenberg, J., & Solomon, S. (2021). Terror Management Theory and the COVID-19 Pandemic. *Journal of Humanistic Psychology*, 61(2), 173-189. <https://doi.org/10.1177/0022167820959488>
