# Supplementary material for: COVID-19 symptoms and compliance: The mediating role of fundamental social motives
Source: Front Psychol. 2023 Mar 20;14:1093875. doi: 10.3389/fpsyg.2023.1093875 (PMC10067610; doi:10.3389/fpsyg.2023.1093875)
Supplement: Supplementary file 1 [file Data_Sheet_1.ZIP › Additional file 3.docx]

**Additional file 3**

**Table S3** Descriptive statistics and bivariate correlations of major variables

| Variables | *M* | *SD* | 95% CI | 1 | | 2 | | 3 | | 4 | | 5 | | | 6 | | 7 | | 8 | | 9 | | 10 | | 11 | |
| --- | --- | --- | --- | --- | --- | --- | --- | --- | --- | --- | --- | --- | --- | --- | --- | --- | --- | --- | --- | --- | --- | --- | --- | --- | --- | --- |
| No symptom (*n* = 12531) | | | | | | | | | | | | | | | | | | | | | | | | | | |
| 1 Compliance | 3.922 | 0.936 | [3.906, 3.939] |  | |  | |  | |  | |  | | |  | |  | |  | |  | |  | |  | |
| 2 Disease avoidance | 4.206 | 0.876 | [4.191, 4.222] | .393^***^ | |  | |  | |  | |  | | |  | |  | |  | |  | |  | |  | |
| 3 Mate retention | 4.130 | 0.891 | [4.115, 4.146] | .445^***^ | | .413^***^ | |  | |  | |  | | |  | |  | |  | |  | |  | |  | |
| 4 Kin care (family) | 4.234 | 0.820 | [4.220, 4.248] | .480^***^ | | .441^***^ | | .563^***^ | |  | |  | | |  | |  | |  | |  | |  | |  | |
| 5 Kin care (children) | 4.144 | 0.969 | [4.127, 4.161] | .424^***^ | | .360^***^ | | .486^***^ | | .604^***^ | |  | | |  | |  | |  | |  | |  | |  | |
| 6 Exclusion concern | 2.996 | 1.014 | [2.978, 3.013] | .073^***^ | | .101^***^ | | .124^***^ | | .107^***^ | | .107^***^ | | |  | |  | |  | |  | |  | |  | |
| 7 Affiliation (group) | 3.223 | 0.971 | [3.206, 3.240] | .228^***^ | | .204^***^ | | .182^***^ | | .164^***^ | | .157^***^ | | | .237^***^ | |  | |  | |  | |  | |  | |
| 8 Affiliation (friendship retention) | 3.788 | 0.899 | [3.772, 3.804] | .343^***^ | | .460^***^ | | .358^***^ | | .368^***^ | | .306^***^ | | | .197^***^ | | .435^***^ | |  | |  | |  | |  | |
| 9 Affiliation (independence) | 2.791 | 1.019 | [2,773, 2.809] | -.004 | | .005 | | -.077^***^ | | -.041^***^ | | -.040^***^ | | | .180^***^ | | .005 | | -.020^*^ | |  | |  | |  | |
| 10 Self-protection | 3.720 | 0.977 | [3.703, 3.737] | .318^***^ | | .529^***^ | | .317^***^ | | .337^***^ | | .284^***^ | | | .152^***^ | | .242^***^ | | .402^***^ | | .059^***^ | |  | |  | |
| 11 Mate seeking | 2.645 | 1.195 | [2.624, 2.666] | .029^**^ | | .030^**^ | | .041^***^ | | .002 | | .014 | | | .260^***^ | | .265^***^ | | .165^***^ | | .101^***^ | | .071^***^ | |  | |
| 12 Status seeking | 3.070 | 1.020 | [3.052, 3.088] | .090^***^ | | .160^***^ | | .123^***^ | | .123^***^ | | .080^***^ | | | .262^***^ | | .360^***^ | | .270^***^ | | .084^***^ | | .183^***^ | | .358^***^ | |
| COVID-19 symptoms (*n* = 687) | | | | | | | | | | | | | | | | | | | | | | | | | |  |
| 1 Compliance | 3.741 | 1.010 | [3.665, 3.817] | |  | |  | |  | |  | |  |  | |  | |  | |  | |  | |  | |  |
| 2 Disease avoidance | 4.052 | 0.940 | [3.982, 4.123] | | .308^***^ | |  | |  | |  | |  |  | |  | |  | |  | |  | |  | |  |
| 3 Mate retention | 3.999 | 0.947 | [3.928, 4.069] | | .382^***^ | | .364^***^ | |  | |  | |  |  | |  | |  | |  | |  | |  | |  |
| 4 Kin care (family) | 4.096 | 0.870 | [4.031, 4.161] | | .413^***^ | | .325^***^ | | .556^***^ | |  | |  |  | |  | |  | |  | |  | |  | |  |
| 5 Kin care (children) | 4.045 | 1.006 | [3.970, 4.120] | | .419^***^ | | .269^***^ | | .482^***^ | | .616^***^ | |  |  | |  | |  | |  | |  | |  | |  |
| 6 Exclusion concern | 3.176 | 0.994 | [3.102, 3.251] | | .130^**^ | | .115^**^ | | .175^***^ | | .167^***^ | | .190^***^ |  | |  | |  | |  | |  | |  | |  |
| 7 Affiliation (group) | 3.204 | 0.935 | [3.134, 3.274] | | .227^***^ | | .172^***^ | | .163^***^ | | .126^**^ | | .179^***^ | .236^***^ | |  | |  | |  | |  | |  | |  |
| 8 Affiliation (friendship retention) | 3.696 | 0.901 | [3.628, 3.763] | | .325^***^ | | .418^***^ | | .409^***^ | | .377^***^ | | .338^***^ | .188^***^ | | .394^***^ | |  | |  | |  | |  | |  |
| 9 Affiliation (independence) | 2.884 | 1.079 | [2.803, 2.964] | | .069 | | -.007 | | -.064 | | -.027 | | -.031 | .271^***^ | | .097^*^ | | -.048 | |  | |  | |  | |  |
| 10 Self-protection | 3.636 | 0.965 | [3.564, 3.708] | | .259^***^ | | .537^***^ | | .298^***^ | | .314^***^ | | .212^***^ | .135^***^ | | .182^***^ | | .399^***^ | | .019 | |  | |  | |  |
| 11 Mate seeking | 2.664 | 1.206 | [2.573, 2.754] | | .076^*^ | | .112^**^ | | .088^*^ | | .047 | | .034 | .243^***^ | | .282^***^ | | .120^**^ | | .225^***^ | | .179^***^ | |  | |  |
| 12 Status seeking | 3.073 | 1.042 | [2.995, 3.151] | | .094^*^ | | .148^***^ | | .065 | | .074 | | .022 | .238^***^ | | .374^***^ | | .197^***^ | | .229^***^ | | .160^***^ | | .374^***^ | |  |
| Other symptoms (*n* = 2540) | | | | | | | | | | | | | | | | | | | | | | | | | |  |
| 1 Compliance | 3.937 | 0.917 | [3.901, 3.973] | |  | |  | |  | |  | |  |  | |  | |  | |  | |  | |  | |  |
| 2 Disease avoidance | 4.158 | 0.874 | [4.124, 4.192] | | .401^***^ | |  | |  | |  | |  |  | |  | |  | |  | |  | |  | |  |
| 3 Mate retention | 4.087 | 0.882 | [4.053, 4.121] | | .407^***^ | | .371^***^ | |  | |  | |  |  | |  | |  | |  | |  | |  | |  |
| 4 Kin care (family) | 4.189 | 0.826 | [4.157, 4.221] | | .465^***^ | | .430^***^ | | .524^***^ | |  | |  |  | |  | |  | |  | |  | |  | |  |
| 5 Kin care (children) | 4.150 | 0.921 | [4.114, 4.186] | | .444^***^ | | .402^***^ | | .490^***^ | | .621^***^ | |  |  | |  | |  | |  | |  | |  | |  |
| 6 Exclusion concern | 3.063 | 1.021 | [3.024, 3.103] | | .111^***^ | | .048^*^ | | .113^***^ | | .113^***^ | | .124^***^ |  | |  | |  | |  | |  | |  | |  |
| 7 Affiliation (group) | 3.219 | 0.956 | [3.181, 3.256] | | .204^***^ | | .180^***^ | | .153^***^ | | .167^***^ | | .148^***^ | .201^***^ | |  | |  | |  | |  | |  | |  |
| 8 Affiliation (friendship retention) | 3.763 | 0.898 | [3.728, 3.798] | | .337^***^ | | .470^***^ | | .336^***^ | | .385^***^ | | .314^***^ | .165^***^ | | .404^***^ | |  | |  | |  | |  | |  |
| 9 Affiliation (independence) | 2.808 | 1.031 | [2.768, 2.848] | | -.002 | | -.024 | | -.077^***^ | | -.058^**^ | | -.058^**^ | .177^***^ | | .046^*^ | | -.013 | |  | |  | |  | |  |
| 10 Self-protection | 3.696 | 0.976 | [3.658, 3.734] | | .333^***^ | | .521^***^ | | .286^***^ | | .320^***^ | | .303^***^ | .122^***^ | | .218^***^ | | .368^***^ | | .029 | |  | |  | |  |
| 11 Mate seeking | 2.602 | 1.193 | [2.555, 2.648] | | .009 | | -.020 | | .009 | | -.026 | | -.027 | .246^***^ | | .256^***^ | | .102^***^ | | .094^***^ | | .035 | |  | |  |
| 12 Status seeking | 3.012 | 1.034 | [2.972, 3.052] | | .101^***^ | | .146^***^ | | .086^***^ | | .089^***^ | | .061^**^ | .248^***^ | | .345^***^ | | .227^***^ | | .112^***^ | | .182^***^ | | .378^***^ | |  |

Note: *^*^p* < 0.05, *^**^p* < 0.01, *^***^p* < 0.001.
