# Supplementary material for: COVID-19 symptoms and compliance: The mediating role of fundamental social motives
Source: Front Psychol. 2023 Mar 20;14:1093875. doi: 10.3389/fpsyg.2023.1093875 (PMC10067610; doi:10.3389/fpsyg.2023.1093875)
Supplement: Supplementary file 1 [file Data_Sheet_1.ZIP › Additional file 4.docx]

**Additional file** **4**

**Table S4** Regression analysis of variable relationships

| Variables | Model 1 | | | | | |
| --- | --- | --- | --- | --- | --- | --- |
|  | **Dependent variable: Compliance (Y)** | | | | | |
|  | ***β*** | ***SE*** | ***t*** | ***p*** | **95% CI** |  |
| Constant | 3.454 | 0.596 | 5.798 | 0.000 | [2.286, 4.622] | *R^2^* = 0.004 |
| COVID-19 symptoms (X1) | -0.037 | 0.037 | -4.644 | 0.000 | [-0.244, -0.099] | *F* = 6.480^***^ |
| Other symptoms (X2) | -0.023 | 0.030 | -1.962 | 0.050 | [-0.119, 0.000] |  |
| Cumulative confirmed cases | 0.006 | 0.086 | 0.788 | 0.431 | [-0.101, 0.237] |  |
| Exposure to infection | -0.031 | 0.090 | -3.849 | 0.000 | [-0.524, -0.170] |  |
| Job status (student) | -0.019 | 0.043 | -2.318 | 0.020 | [-0.185, -0.015] |  |
| Job status (retiree) | -0.026 | 0.030 | -2.782 | 0.005 | [-0.140, -0.024] |  |
| Age | -0.001 | 0.001 | -0.054 | 0.957 | [-0.001, 0.001] |  |
| Gender | 0.005 | 0.016 | 0.604 | 0.546 | [-0.022, 0.042] |  |
| Disease history | 0.008 | 0.020 | 0.886 | 0.376 | [-0.022, 0.058] |  |
| Symptom description | 0.035 | 0.038 | 2.931 | 0.003 | [0.037, 0.187] |  |
| Medical staff | 0.014 | 0.048 | 1.731 | 0.083 | [-0.011, 0.178] |  |
| Pregnant | 0.021 | 0.116 | 2.536 | 0.011 | [0.067, 0.523] |  |

| Variables | Model 2 | | | | | | Model 3 | | | | | |
| --- | --- | --- | --- | --- | --- | --- | --- | --- | --- | --- | --- | --- |
|  | **Dependent variable: M** | | | | | | **Dependent variable: Compliance (Y)** | | | | | |
|  | ***β*** | ***SE*** | ***t*** | ***p*** | **95% CI** |  | ***β*** | ***SE*** | ***t*** | ***p*** | **95% CI** |  |
| Constant | 3.920 | 0.559 | 7.015 | 0.000 | [2.825, 5.016] | *R^2^*= 0.005 | 1.828 | 0.550 | 3.326 | 0.001 | [0.751, 2.906] | *R^2^*= 0.155 |
| COVID-19 symptoms (X1) | -0.033 | 0.035 | -4.061 | 0.000 | [-0.209, -0.073] | *F* = 7.451^***^ | -0.025 | 0.034 | -3.324 | 0.001 | [-0.180, -0.046] | *F* = 223.102^***^ |
| Other symptoms (X2) | -0.053 | 0.029 | -4.446 | 0.000 | [-0.183, -0.071] |  | -0.003 | 0.028 | -0.251 | 0.801 | [-0.062, 0.048] |  |
| Disease avoidance |  |  |  |  |  |  | 0.389 | 0.008 | 52.997 | 0.000 | [0.399, 0.430] |  |
| Constant | 4.661 | 0.565 | 8.244 | 0.000 | [3.553, 5.769] | *R^2^* = 0.012 | 1.315 | 0.537 | 2.447 | 0.014 | [0.262, 2.368] | *R^2^*= 0.193 |
| COVID-19 symptoms (X1) | -0.032 | 0.035 | -3.943 | 0.000 | [-0.207, -0.069] | *F* = 16.844^***^ | -0.024 | 0.033 | -3.249 | 0.001 | [-0.173, -0.043] | *F* = 290.944^***^ |
| Other symptoms (X2) | -0.057 | 0.029 | -4.773 | 0.000 | [-0.194, -0.081] |  | 0.001 | 0.027 | 0.130 | 0.896 | [-0.050, 0.057] |  |
| Mate retention |  |  |  |  |  |  | 0.437 | 0.008 | 60.715 | 0.000 | [0.444, 0.474] |  |
| Constant | 4.791 | 0.524 | 9.149 | 0.000 | [3.765, 5.818] | *R^2^* = 0.005 | 0.875 | 0.526 | 1.662 | 0.096 | [-0.157, 1.906] | *R^2^*= 0.227 |
| COVID-19 symptoms (X1) | -0.034 | 0.032 | -4.268 | 0.000 | [-0.202, -0.075] | *F* = 7.604^***^ | -0.021 | 0.033 | -2.977 | 0.003 | [-0.161, -0.033] | *F* = 357.225^***^ |
| Other symptoms (X2) | -0.060 | 0.027 | -5.063 | 0.000 | [-0.188, -0.083] |  | 0.005 | 0.027 | 0.492 | 0.622 | [-0.039, 0.066] |  |
| Kin care (family) |  |  |  |  |  |  | 0.473 | 0.008 | 67.407 | 0.000 | [0.523, 0.554] |  |
| Constant | 3.458 | 0.602 | 5.743 | 0.000 | [2.278, 4.638] | *R^2^* = 0.037 | 1.979 | 0.538 | 3.678 | 0.000 | [0.925, 3.034] | *R^2^* = 0.189 |
| COVID-19 symptoms (X1) | -0.025 | 0.037 | -3.208 | 0.001 | [-0.193, -0.047] | *F* = 51.264^***^ | -0.026 | 0.033 | -3.613 | 0.000 | [-0.186, -0.055] | *F* = 283.713^***^ |
| Other symptoms (X2) | -0.043 | 0.031 | -3.674 | 0.000 | [-0.173, -0.053] |  | -0.005 | 0.027 | -0.419 | 0.675 | [-0.065, 0.042] |  |
| Kin care (children) |  |  |  |  |  |  | 0.438 | 0.007 | 59.940 | 0.000 | [0.413, 0.440] |  |
| Constant | 3.322 | 0.645 | 5.148 | 0.000 | [2.057, 4.586] | *R^2^* = 0.005 | 3.203 | 0.594 | 5.390 | 0.000 | [2.038, 4.368] | *R^2^*= 0.011 |
| COVID-19 symptoms (X1) | 0.038 | 0.040 | 4.747 | 0.000 | [0.111, 0.268] | *F* = 7.920^***^ | -0.040 | 0.037 | -5.045 | 0.000 | [-0.258, -0.114] | *F* = 14.176^***^ |
| Other symptoms (X2) | 0.044 | 0.033 | 3.645 | 0.000 | [0.056, 0.185] |  | -0.027 | 0.030 | -2.266 | 0.023 | [-0.128, -0.009] |  |
| Exclusion concern |  |  |  |  |  |  | 0.082 | 0.007 | 10.296 | 0.000 | [0.061, 0.090] |  |
| Constant | 2.207 | 0.614 | 3.592 | 0.000 | [1.003, 3.410] | *R^2^* = 0.007 | 2.976 | 0.581 | 5.123 | 0.000 | [1.837, 4.115] | *R^2^* = 0.054 |
| COVID-19 symptoms (X1) | -0.001 | 0.038 | -0.068 | 0.945 | [-0.077, 0.072] | *F* = 9.923^***^ | -0.037 | 0.036 | -4.749 | 0.000 | [-0.241, -0.100] | *F* = 69.915^***^ |
| Other symptoms (X2) | 0.009 | 0.031 | 0.713 | 0.476 | [-0.039, 0.084] |  | -0.025 | 0.030 | -2.176 | 0.030 | [-0.123, -0.006] |  |
| Affiliation (group) |  |  |  |  |  |  | 0.224 | 0.008 | 28.758 | 0.000 | [0.202, 0.231] |  |
| Constant | 4.612 | 0.572 | 8.067 | 0.000 | [3.492, 5.733] | *R^2^* = 0.003 | 1.812 | 0.561 | 3.229 | 0.001 | [0.712, 2.911] | *R^2^*= 0.120 |
| COVID-19 symptoms (X1) | -0.018 | 0.035 | -2.178 | 0.029 | [-0.147, -0.008] | *F* = 5.204^***^ | -0.031 | 0.035 | -4.149 | 0.000 | [-0.212, -0.076] | *F* = 166.957^***^ |
| Other symptoms (X2) | -0.004 | 0.029 | -0.297 | 0.767 | [-0.066, 0.049] |  | -0.022 | 0.029 | -1.979 | 0.048 | [-0.113, -0.001] |  |
| Affiliation (friendship retention) |  |  |  |  |  |  | 0.342 | 0.008 | 45.633 | 0.000 | [0.341, 0.371] |  |
| Constant | 3.339 | 0.650 | 5.137 | 0.000 | [2.065, 4.614] | *R^2^* = 0.006 | 3.451 | 0.596 | 5.788 | 0.000 | [2.283, 4.620] | *R^2^* = 0.004 |
| COVID-19 symptoms (X1) | 0.022 | 0.040 | 2.726 | 0.006 | [0.031, 0.189] | *F* = 8.916^***^ | -0.037 | 0.037 | -4.645 | 0.000 | [-0.244, -0.099] | *F* = 5.982^***^ |
| Other symptoms (X2) | 0.031 | 0.033 | 2.597 | 0.009 | [0.021, 0.151] |  | -0.023 | 0.030 | -1.964 | 0.050 | [-0.119, 0.000] |  |
| Affiliation (independence) |  |  |  |  |  |  | 0.001 | 0.007 | 0.112 | 0.911 | [-0.013, 0.015] |  |
| Constant | 4.036 | 0.622 | 6.492 | 0.000 | [2.817, 5.254] | *R^2^*= 0.002 | 2.229 | 0.566 | 3.939 | 0.000 | [1.120, 3.338] | *R^2^*= 0.104 |
| COVID-19 symptoms (X1) | -0.014 | 0.039 | -1.799 | 0.072 | [-0.145, 0.006] | *F* = 2.984^**^ | -0.033 | 0.035 | -4.295 | 0.000 | [-0.219, -0.082] | *F* = 141.814^***^ |
| Other symptoms (X2) | -0.010 | 0.032 | -0.838 | 0.402 | [-0.089, 0.036] |  | -0.020 | 0.029 | -1.788 | 0.074 | [-0.108, 0.005] |  |
| Self-protection |  |  |  |  |  |  | 0.316 | 0.007 | 41.918 | 0.000 | [0.289, 0.318] |  |
| Constant | 2.570 | 0.751 | 3.423 | 0.001 | [1.098, 4.042] | *R^2^* = 0.028 | 3.397 | 0.596 | 5.702 | 0.000 | [2.230, 4.565] | *R^2^* = 0.005 |
| COVID-19 symptoms (X1) | 0.011 | 0.047 | 1.410 | 0.159 | [-0.026, 0.157] | *F* = 38.339^***^ | -0.038 | 0.037 | -4.685 | 0.000 | [-0.245, -0.101] | *F* = 6.928^***^ |
| Other symptoms (X2) | 0.027 | 0.038 | 2.320 | 0.020 | [0.014, 0.164] |  | -0.024 | 0.030 | -2.027 | 0.043 | [-0.121, -0.002] |  |
| Mate seeking |  |  |  |  |  |  | 0.028 | 0.006 | 3.499 | 0.000 | [0.010, 0.035] |  |
| Constant | 4.468 | 0.633 | 7.059 | 0.000 | [3.227, 5.708] | *R^2^* = 0.057 | 3.064 | 0.594 | 5.156 | 0.000 | [1.899, 4.228] | *R^2^* = 0.013 |
| COVID-19 symptoms (X1) | 0.011 | 0.039 | 1.398 | 0.162 | [-0.022, 0.132] | *F* = 80.281^***^ | -0.038 | 0.037 | -4.794 | 0.000 | [-0.248, -0.104] | *F* = 16.572^***^ |
| Other symptoms (X2) | 0.014 | 0.032 | 1.212 | 0.226 | [-0.024, 0.103] |  | -0.025 | 0.030 | -2.083 | 0.037 | [-0.123, -0.004] |  |
| Status seeking |  |  |  |  |  |  | 0.095 | 0.007 | 11.705 | 0.000 | [0.073, 0.102] |  |

Note: Gender (male = 0, female = 1); *β* = standardized beta; CI = confidence intervals; *^*^p* < 0.05, *^**^p* < 0.01, *^***^p* < 0.001.
